# HMGN2 regulates non‐tuberculous mycobacteria survival via modulation of M1 macrophage polarization

**DOI:** 10.1111/jcmm.14599

**Published:** 2019-10-09

**Authors:** Xinyuan Wang, Shanze Chen, Hongyu Ren, Junli Chen, Jingyu Li, Yi Wang, Yuanqi Hua, Xiaoying Wang, Ning Huang

**Affiliations:** ^1^ Department of Pathophysiology, West China School of Basic Medical Sciences and Forensic Medicine Sichuan University Chengdu China; ^2^ Comprehensive Pneumology Center (CPC), University Hospital, Ludwig-Maximilians University Helmholtz Zentrum München, Member of the German Center for Lung Research (DZL) Munich Germany

**Keywords:** HMGN2, IFNγ, macrophage polarization, non‐tuberculous mycobacteria

## Abstract

Non‐tuberculous mycobacteria (NTM), also known as an environmental and atypical mycobacteria, can cause the chronic pulmonary infectious diseases. Macrophages have been suggested as the main host cell to initiate the innate immune responses to NTM infection. However, the molecular mechanism to regulate the antimicrobial immune responses to NTM is still largely unknown. Current study showed that the NTM clinical groups, *Mycobacterium abscessus* and *Mycobacterium smegmatis,* significantly induced the M1 macrophage polarization with the characteristic production of nitric oxide (NO) and marker gene expression of iNOS, IFNγ, TNF‐α, IL1‐β and IL‐6. Interestingly, a non‐histone nuclear protein, HMGN2 (high‐mobility group N2), was found to be spontaneously induced during NTM‐activated M1 macrophage polarization. Functional studies revealed that HMGN2 deficiency in NTM‐infected macrophage promotes the expression of M1 markers and the production of NO via the enhanced activation of NF‐κB and MAPK signalling. Further studies exhibited that HMGN2 knock‐down also enhanced IFNγ‐induced M1 macrophage polarization. Finally, we observed that silencing HMGN2 affected the survival of NTM in macrophage, which might largely relevant to enhanced macrophage polarization into M1 phenotype under the NTM infection. Collectively, current studies thus suggested a novel function of HMGN2 in regulating the anti‐non‐tuberculous mycobacteria innate immunity of macrophage.

## 1 INTRODUCTION

Non‐tuberculous mycobacteria (NTM), as an environmental opportunistic mycobacteria, are different from *Mycobacterium tuberculosis* (TB) but still could cause disease in human, such as pulmonary infections, lymphadenitis, skin and soft tissue infections.^1^, ^2^, ^3^, ^4^ It is a group of conditional pathogen cause even more diseases burden than TB. In recent years, NTM infection is increasing quickly over worldwide and becomes a very important global public health problem.^5^, ^6^, ^7^ And in America, more than 90% of NTM isolation strains are from pulmonary secretions.^8^

It has been well noted that macrophages are the host primary cells, macrophage can destroy mycobacteria or be a reservoir for intracellular non‐tuberculous mycobacteria replication.^9^ In general, to fight against non‐tuberculous mycobacteria infection, macrophage can initiate innate immunity though recognition of pathogen‐associated molecular patterns (PAMP) by pattern‐recognition receptors (PRRs),^10^ such as activation of phagocytic pathway, production of nitric oxide (NO) and antibacterial peptide,^11^, ^12^, ^13^, ^14^ release of pro‐inflammatory cytokines and chemokines,^12^, ^15^, ^16^ which was conceptually introduced as classical activation of macrophage, also termed as M1 polarization.^17^ Following the acute inflammatory burst, however, macrophages can remove cell debris and apoptotic inflammatory cells by so‐called efferocytosis and polarize into alternatively activated, M2 macrophages.^18^

It has been well documented that M1 macrophage polarization is instructed by a variety of environmental stimuli, for instance the acute infection of bacteria and virus, or the stimulation of LPS and IFNγ. The activated macrophages lead to the pro‐inflammatory responses and further limit bacterial growth. Currently, it has been widely studied about the immune response against *Mycobacterium tuberculosis*. For instance, in the initial infection stage, *Mycobacterium tuberculosis* subcellular components activated M1 macrophage to produce pro‐inflammatory cytokines such as tumour necrosis factor alpha (TNF‐α), IL‐1β, IL‐6, IL‐12, nitric oxide (NO), reactive oxygen species (ROS), and chemokines via TLR2 signalling.^19^ In NTM infection model, it has been reported that NTM‐induced different cytokine patterns depend on the strains of NTM which is related to the intracellular NTM growth rate.^20^, ^21^

IFNγ is the only subunit of type II interferons. It has been well noted that IFNγ is not only an essential inducer of the M1 macrophage polarization, but also plays the crucial role in anti‐*Mycobacterium tuberculosis* defence.^22^ It has been reported that IFNγ promotes NO production to further induce iNOS transcription, which increased the ability of macrophage clearance intracellular pathogens such as the *Mycobacterium tuberculosis*.^23^ The expression of IFNγ could be induced by *Mycobacterium tuberculosis* in macrophage. In addition, it was reported that IFNγ been up‐regulated by released cytokines. For example, IL‐12 and TNF‐α can induce IFNγ expression.^24^, ^25^, ^26^ All in all, tight regulation of cellular cytokine pathways is critical to shape the macrophage polarization state and further impact on the final results of the host anti‐bacterial immune responses. Therefore, it is necessary and important to explore the regulatory mechanism of inflammatory cytokine expression and the function of macrophages during mycobacteria infection.

High‐mobility group (HMG) is a group of chromosomal proteins which are found in the mammalian nuclei. HMGN serves as a member of the HMG super family, which is involved in the regulation of gene transcription, replication, recombination and DNA repair.^27^ HMGN protein family is composed of five subtypes: HMGN1, HMGN2, HMGN3, HMGN4 and HMGN5. Since 2004, we firstly identified HMGN2 functioned as an antimicrobial peptides,^28^ and furthermore, we have demonstrated that HMGN2 is a multifunctional protein in immune regulation. For example, we found that HMGN2 promotes LPS‐induced β‐defensin expression in lung epithelial cells.^29^ HMGN2 inhibits the internalization of *Klebsiella pneumoniae* in lung epithelial cell A549 through decreasing the expression and activity of α5β1 integrin and activated FAK‐Src signalling to reduce F‐actin polymerization.^30^ HMGN2 was found to be used to through Nrf‐2 signalling prevents *Pseudomonas aeruginosa* adhesion and invasion in lung epithelial cells by promoting pyocyanin‐induced intracellular ROS clearance.^31^ Otherwise, we found HMGN2 participated in MAPK signalling through activating ERK1/2 and P38, and regulating autophagy by AMPK pathway to reduce *Uropathogenic Escherichia coli* internalization of bladder epithelial cells.^32^ However, the role of HMGN2 for regulating the anti‐non‐tuberculous mycobacteria innate immunity of macrophage is still largely unknown.

In the current study, we firstly investigated the macrophage polarization potential upon the two strains of non‐tuberculous mycobacteria infection, and then we show the expression pattern of HMGN2 in macrophages under different infection conditions. Furthermore, we detected HMGN2 functional relevance and its effect on M1‐related signalling pathways in M1 macrophage by using the RNA interfering technology. Lastly, we investigated the expression pattern of HMGN2 under IFNγ stimulation and the role of HMGN2 in IFNγ promoted M1 macrophage polarization.

## 2 MATERIALS AND METHODS

### 2.1 Reagent and antibodies

Rabbit monoclonal antibody for HMGN2 was provided by Cell Signaling Technology. Rabbit polyclonal antibodies for iNOS, phospho‐JNK, JNK, phospho‐P38, P38, phospho‐ERK1/2, ERK1/2, phospho‐IκBα and IκBα were provided by Signalway Antibody. Phospho‐P65, rabbit polyclonal antibody for P65 and rabbit monoclonal antibody for phospho‐P65 were purchased from Beyotime Institute of Biotechnology. FITC fluorescent‐labelled secondary antibody (goat anti‐rabbit IgG, green) and tetramethylrhodamine (TRITC)‐conjugated secondary antibody (goat anti‐rabbit IgG, red) were also purchased from Beyotime Institute of Biotechnology. DAPI and FITC were provided by Sigma‐Aldrich. All‐in‐One cDNA Synthesis SuperMix and 2xSYBR Green RT‐qPCR Master Mix were purchased from Biotool. Selective IKK inhibitor BMS‐345541 was obtained from Selleck. RPMI 1640 medium was purchased from HyClone, Thermo Scientific. Foetal bovine serum (FBS) was obtained from FuMeng Gene Co., Ltd.. Penicillin‐streptomycin was purchased from Beijing Solarbio Science and Technology Co., Ltd.. IFNγ was purchased from immune tool. Other chemical reagents were all analytical grade.

### 2.2 Microbial strain and culture condition

*Mycobacterium abscessus24* (*Ma.24*) and *Mycobacterium smegmatis13* (*Ms13*) were isolated from a sputum sample which was obtained from respiratory infection patients. Mycobacteria strains were identified by sequencing and using sequencing of housekeeping gene target hsp65 primer (sense: 5′‐ATCGCCAAGGAGATCGAGCT‐3′, anti‐sense: 5′‐AAGGTGCCGCGGATCTTGTT‐3′). *Ma.24* and *Ms13* were frozen at −80°C and grown at 37°C on Middle Brook 7H11 Agar solid culture medium for 4‐5 days.

### 2.3 Cell line and cell culture

Mus musculus macrophage cell lines RAW264.7 and MH‐S were purchased from the Cell Bank of the Chinese Academic of Sciences. RAW264.7 cells were cultured in DMEM high glucose medium (HyClone) with 10% foetal bovine serum which be pre‐incubated at 56°C for 30 minutes (FBS, FuMeng Gene Co.,Ltd.) and antibiotics (100 U/mL penicillin and 100 µg/mL streptomycin). Cells were incubated in humidified air with 5% CO_2_ at 37°C. MH‐S cells were cultured in RPMI‐1640 medium supplemented with 10% foetal bovine serum and 50 µmol/L β‐mercaptoethanol and antibiotics (100 U/mL penicillin and 100 µg/mL streptomycin incubated in humidified air with 5% CO_2_ at 37°C.

### 2.4 RNA interference

Mouse HMGN2‐specific small interfering RNA (siRNA‐HMGN2, target sequence: GACGAGCCACAGAGAAGAT) and negative control siRNA (siRNA‐NC) were synthesized by RiboBio Co. Ltd. Silencing experiments were performed by siRNA‐HMGN2 (final concentration 2 nmol/L) and siRNA‐NC with same concentration. siRNA and opti‐MEM were mixed for 5 minutes at RT. The INTERFERin (Polyplus) was added into opti‐MEM and incubated for another 20 minutes at RT and then added to the cells.

### 2.5 Cell viability assay

The in vitro cell viability of the macrophage lines was assessed by the Cell Counting Kit‐8 (CCK‐8, Vazyme Biotech). RAW264.7 and MH‐S cells were exposed to *Ma.24* and *Ms.13* separately at an MOI of 10:1 for 12 and 24 hours. For siHMGN2 transfected RAW264.7 and MH‐S cells, pre‐transfected with siRNA for 24 hours and then incubated with *Ma.24* and *Ms.13* separately at an MOI of 10:1 for 12 and 24 hours. A total of 10 µL of CCK‐8 solution was added to each well. Cells were cultured for an additional 3 hours and measured by a microplate reader (BioTek) at 450 nm.

### 2.6 Real‐time quantitative polymerase chain reaction (RT‐QPCR)

Total RNA from RAW264.7 and MH‐S cells was extracted by UNIQ‐10 Column total RNA Purification Kit (Sangon Biotech) following the manufacturer's instruction. The purity and concentration of total RNA were measured by Implen NanoPhotometer. The cDNA synthesis was achieved using All‐in‐One cDNA Synthesis SuperMix (Biotool). The PCR was performed with CFX96 Real‐Time PCR, and the PCR products were detected using 2xSYBR Green RT‐qPCR Master Mix (Biotool). The PCR primers were synthesized by Qinke, and the primer sequences were as follows: HMGN2, TNF‐α, IFNγ, IL‐1β, IL‐6, IL‐10, TGF‐β, iNOS, LL37, IFITM1, CXCL1 and CXCL2. The relative mRNA transcripts levels in different groups were evaluated using the 2^−ΔΔ^ *^C^* ^t^ methods (Table 1).

**Table 1:** RT‐qPCR Primer Sequences (5'‐3')

Target gene	Forward primer (5′‐3′)	Reverse primer (5′‐3′)
*Actb*	TCCATCATGAAGTGTGACGT	GAGCAATGATCTTGATCTTCAT
HMGN2	TGAAGGGGATGCTAAAGGAGA	GTGCCTGGTCTGTTTTGGC
iNOS	CCT GTG AGA CCT TTG ATG	CCTATATTGCTGTGGCTC
TNF‐ α	CACCACGCTCTTCTGTCT	GGCTACAGGCTTGTCACTC
IL‐1β	CAACCAACAAGTGATATTCTCCATG	GATCCACACTCTCCAGCTGCA
IL‐6	TAGTCCTTCCTACCCCAATTTCC	TTGGTCCTTAGCCACTCCTTC
IL‐10	GCTGGACAACATACTGCTAACC	ATTTCCGATAAGGCTTGGCAA
TGF‐β	TGACGTCACTGGAGTTGTACG	GGTTCATGTCATGGATGGTGC
IFNγ	ACGGCACAGTCATTGAAAGCCTA	GTCACCATCCTTTTGCCAGTTCC
LL37	GCTGTGGCGGTCACTATCAC	TGTCTAGGGACTGCTGGTTGA
IFITM1	GAAGATGGTGGGTGATACGA	GCAGCGATAGACAAGGAAAC
CXCL1	CCGAAGTCATAGCCACAC	GTGCCATCAGAGCAGTCT
CXCL2	TCCAGAGCTTGAGTGTGACG	TCCAGGTCAGTTAGCCTTGC
YM1	CAGGTCTGGCAATTCTTCTGAA	GTCTTGCTCATGTGTGTAAGTGA
Arg1	CTCCAAGCCAAAGTCCTTAGAG	AGGAGCTGTCATTAGGGACATC
CXCR2	GCCCTGCCCATCTTAATTCTAC	ACCCTCAAACGGGATGTATTGT

### 2.7 Western blotting assay

RAW264.7 and MH‐S cells were plated in 12‐well plate. Collected the cell pellet and extracted proteins with RIPA buffer. The cell lysates were centrifuged at 14 000 × *g* for 20 minutes at 4°C. The total protein concentration was determined by BCA assay kit (KeyGen Biotech). Equal amounts of 20 µg protein lysate were added into the wells of the SDS‐PAGE gels. The separated cells were then transferred onto a PVDF membrane and blocked with 5% blotting milk in PBS buffer for 1 hour, and incubated with the diluted primary antibody in 0.5% PBST buffer overnight at 4°C, then added the secondary antibody (1:1000) for 2 hours at room temperature. The intensity of the band signals were detected by enhanced chemiluminescence (Merck Millipore) and exposed with ChemiDoc™ MP Imager (Bio‐Rad).

### 2.8 Immunofluorescence microscopy

The RAW264.7 and MH‐S cells were plated on the glass slide. The slides were washed with PBS and then fixed with 4% paraformaldehyde for 20 minutes at room temperature and permeabilized with 0.5% Triton X‐100 for 15 minutes washed with PBS three times before blocking in 5% BSA for 1 hour. The slides incubated with first antibodies at 4°C overnight and then incubated with secondary antibody for 1 hour in RT. Nucleus was stained with 4′,6‐diamidino‐2‐phenylindole (DAPI) for 5 minutes. *Ma.24* and *Ms13* were stained with FITC for 1 hour. The stained slides were observed under the confocal immunofluorescence microscope (Olympus FV‐10000).

### 2.9 NO assay

The production of NO in macrophages was determined by the Total Nitric Oxide Assay Kit (Beyotime) according to the manufacturer's instructions. Briefly, total NO production was measured by Griess reagent to react with NO metabolite nitrite in the medium to form a coloured product and then quantified by a microplate reader at 540 nm.

### 2.10 Intracellular bacterial assay

According to our previous method,^30^ macrophages were plated with 5 × 10^5^ into a 24‐well plate and cultured for 24 hours to adhere. The treated cells were washed with PBS to remove antibiotics and then infected with NTM at a multiplicity of infection (MOI) of 10:1 for 2 hours at 37°C. For the number of bacterial been engulfed by macrophage, cells were washed with PBS and add 0.25% Triton X‐100 to lyse infected macrophage. After a serial dilution of lysed medium, bacteria were plated onto Middle Brook 7H11 Agar solid culture medium at 37°C. The colony‐forming units (CFU) of bacteria were counted after 3 days. For the number of survival bacterial in macrophage, after 2 hours infection, cells were incubated with 100 µg/mL gentamicin in cell culture medium at 37°C for 2 hours to kill the extracellular bacteria and then cultured the cells for 3‐12 hours to detect intracellular bacteria by counting CFU as mentioned above.

### 2.11 Statistical analysis

Data were analysed by two‐tailed Student's *t* test to compare the differences in values between experienced group and control group. *P*‐value < .05 was considered to have a statistical difference.

## 3 RESULTS

### 3.1 Non‐tuberculous mycobacteria stimulated macrophage M1 polarization

**Figure 1:**
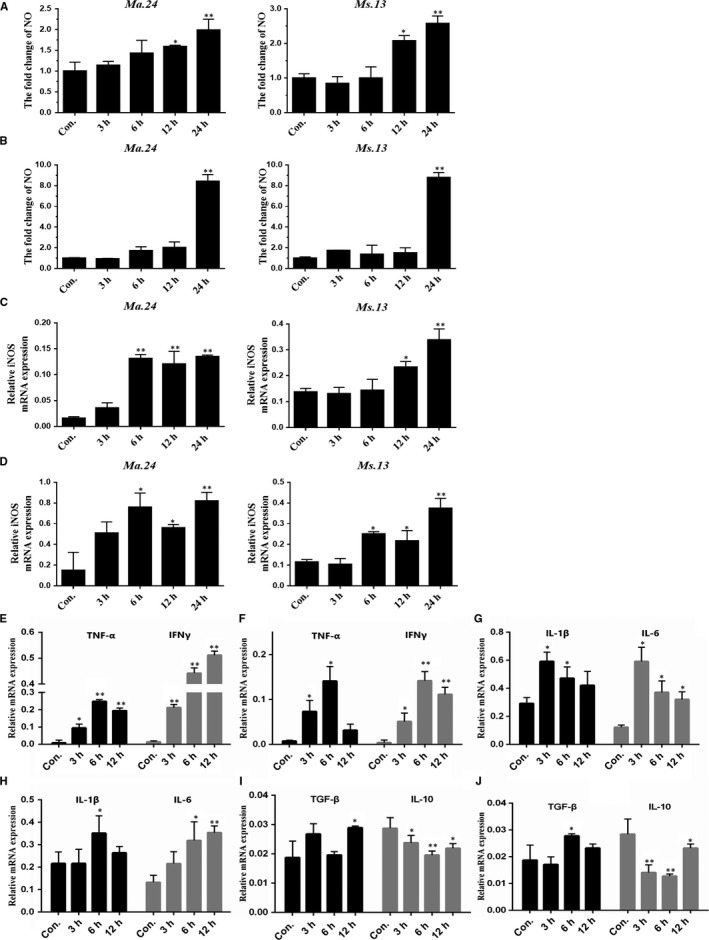
Non‐tuberculous mycobacteria infection induces M1 macrophage polarization. RAW264.7 and MH‐S cells were exposed to NTM *Ma. 24* and *Ms 13* separately (MOI = 10:1) at indicated time courses 3, 6, 12 and 24 h. Results shown are representative of at least three independent experiments. A and B, The production of NO was examined by nitrate reduction method. *P* value was determined by one sample *t* test. *Indicates significant difference (*P < .05; **P < .01) between uninfected and infected groups. C and D, The expression levels of iNOS transcription were determined by RT‐qPCR *P* value was determined by one sample *t* test. *Indicates significant difference (*P < .05; **P < .01) between uninfected and infected groups. RAW264.7 was exposed to *Ma. 24* (E, G and I) and *Ms 13* (F, H and J) separately (MOI = 10:1) at indicated time‐points 3, 6 and 12 h. Results shown are representative of at least three independent experiments. Pro‐inflammatory cytokines TNF‐α, IFNγ (E and F), IL‐1β and IL‐6(G and H), and anti‐inflammatory cytokines TGF‐β, IL‐10 (I and J) mRNA transcription expression were examined by RT‐qPCR *P* value was determined by one sample *t* test. *Indicates significant difference (*P < .05; **P < .01) between uninfected and infected groups

*Mycobacterium abscessus 24* (*Ma.24*) and *Mycobacterium smegmatis 13* (*Ms13*) are two non‐tuberculous mycobacteria strains isolated from clinical chronic pulmonary patients. To explore the early reaction of macrophage infected by these two NTM strains, we performed NO assay via incubating macrophage RAW264.7 and MH‐S cells with *Ma.24* (MOI = 10:1) and *Ms13* (MOI = 10:1) separately. The cytotoxicity of macrophages induced by non‐tuberculous mycobacteria strains was tested by CCK‐8(Figure S1). Our results showed that NO was induced at 12 hours in RAW264.7 (Figure 1A) and 24 hours in MH‐S cells (Figure 1B). Then, we measured the expression of iNOS in NTM‐infected macrophages. iNOS mRNA was increased in a time course–dependent manner (Figure 1C and D). Meanwhile, we investigated the expression pattern of TNF‐α, IFNγ, IL‐1β and IL‐6 which are crucial pro‐inflammatory cytokines to involve in the early stage of immune responses and are also well used for indication macrophage M1 polarization status. We observed that the maximal transcription of TNF‐α at 6 hours in *Ma.24‐* or *Ms13*‐infected RAW264.7 cells. IFNγ was induced by *Ma.24* infection for 37.3‐fold at 12 hours (Figure 1E and F). Interestingly, the increased peak of IL‐1β and IL‐6 in *Ma.24*‐infected RAW264.7 was earlier than *Ms13* infection at 3 hours (Figure 1G and H). Anti‐inflammatory cytokine IL‐10 mRNA was decreased from 3 hours, and TGF‐β was lightly increased after infection (Figure 1I and J). Therefore, our results indicated that non‐tuberculous mycobacteria strains *Ma.24* and *Ms13* could stimulate macrophage M1 polarization in the early stage of NTM infection with different sensitivity.

### 3.2 HMGN2 was induced in non‐tuberculous mycobacteria‐infected macrophage

**Figure 2:**
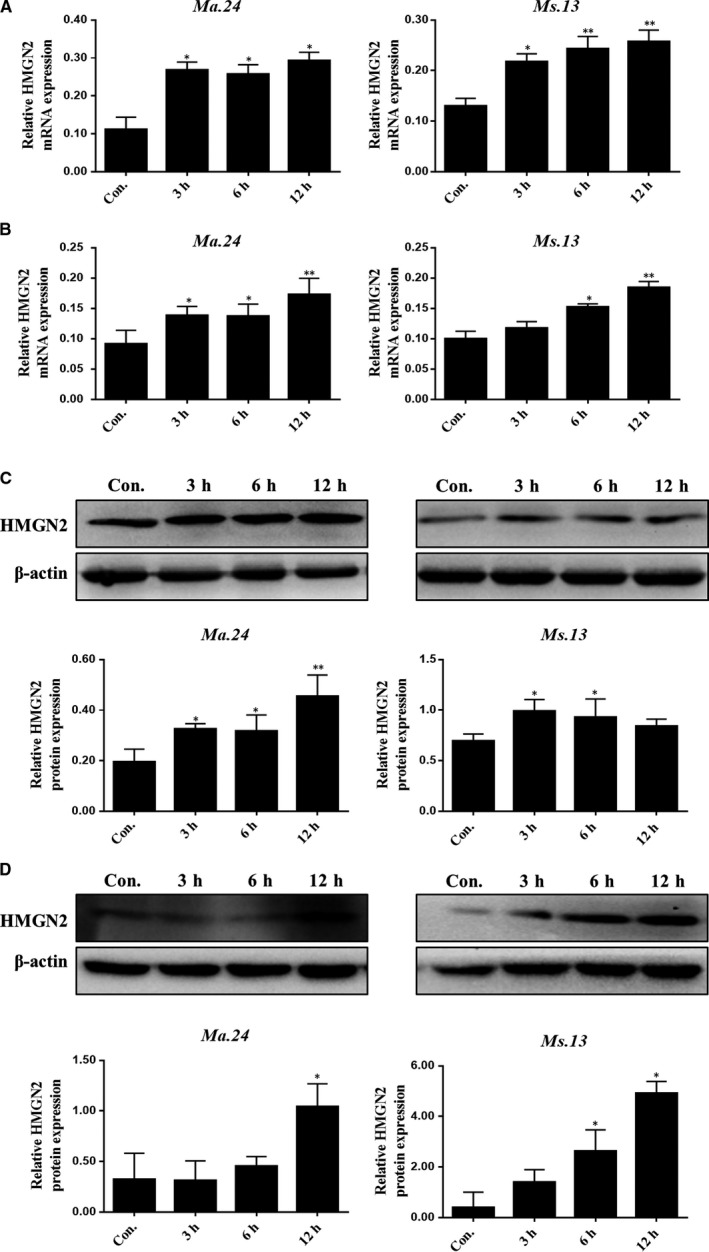
HMGN2 is up‐regulated in NTM‐polarized M1 macrophage. RAW264.7 and MH‐S cells were exposed to *Ma. 24* and *Ms 13* separately (MOI = 10:1) at indicated time‐points 3, 6 and 12 h. Results shown are representative of three independent experiments. A and B, RT‐qPCR examined HMGN2 transcription in infected macrophages. *P* value was determined by one sample *t* test. *Indicates significant difference (*P < .05; **P < .01) between uninfected and infected groups. C and D, Western blot analysis showed the effect of non‐tuberculosis mycobacteria infection on HMGN2 expression. Quantification was measured by Image J, and *P* value was determined by one sample *t* test. *Indicates significant difference (*P < .05; **P < .01) between uninfected and infected groups

Our previous studies showed that HMGN2 was up‐regulated in bladder epithelial cells induced by *Uropathogenic Escherichia coli* in vivo and in vitro. We further assessed whether the expression of HMGN2 was changed in polarized M1 macrophages by the use of quantitative RT‐qPCR and Western blotting assay after time‐dependent NTM infection. Macrophage RAW264.7 and MH‐S cells infected with *Ma.24* (MOI = 10:1) and *Ms13* (MOI = 10:1) separately in a time courses infection. The mRNA level of HMGN2 expression was higher from 3 to 12 hours (Figure 2A and B). And the protein level of HMGN2 was significantly higher than the uninfected group (Figure 2C and D). In addition, we examined the cellular distribution of HMGN2 in macrophage. Immunofluorescence assay showed that HMGN2 localized in both cytoplasm and nucleus, and NTM infection does not alter HMGN2 distribution (Figure S2A).

### 3.3 HMGN2 knock‐down enhanced M1 macrophage polarization with characteristic increase of NO production and the up‐regulation of iNOS

**Figure 3:**
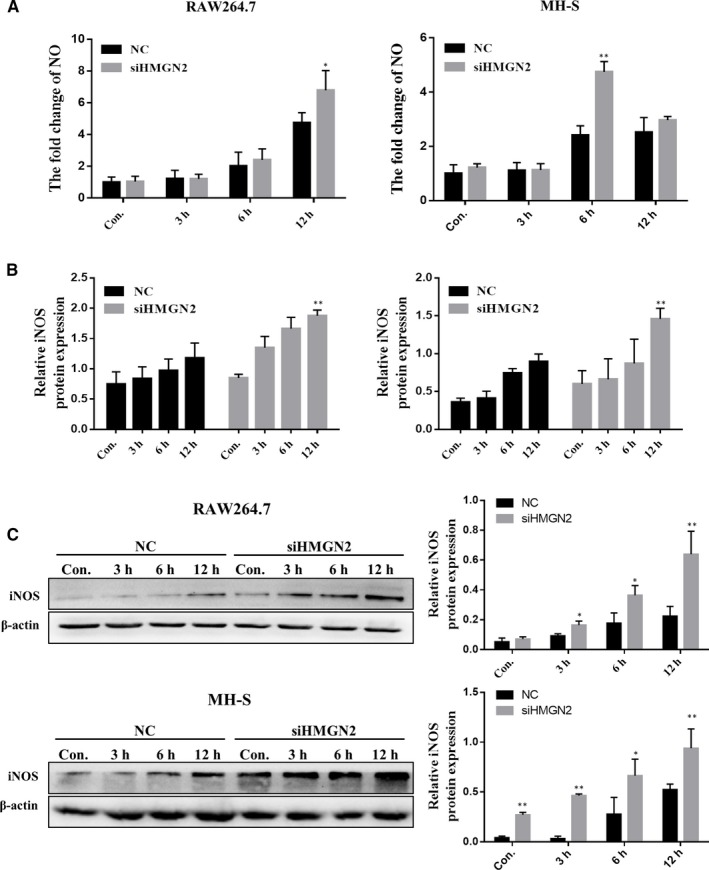
HMGN2 knock‐down enhances NTM‐induced M1 macrophage polarization. RAW264.7 cells were transfected with siRNA‐HMGN2 and NC siRNA (scramble control) for 24 h and then incubated with *Ma. 24* (MOI = 10:1) for 3, 6 and 12 h. A, The production of NO was examined by nitrate reduction method. *P* value was determined by one sample *t* test. *Indicates significant difference (*P < .05; **P < .01) between NC‐infected and siHMGN2‐infected groups. Results shown are representative of at least three independent experiments. B, The expression level of iNOS was determined by RT‐qPCR *P* value was determined by one sample *t* test. *Indicates significant difference (**P < .01) between NC‐infected and siHMGN2‐infected groups at the same time‐point. C, Western blot analysis showed the protein level of iNOS expression. *P* value was determined by one sample *t* test. *Indicates significant difference (*P < .05; **P < .01) between NC‐infected and siHMGN2‐infected groups at the same time‐point

iNOS, an inducible enzyme to generate the nitric oxide for killing pathogens, is well described to characterize the M1 polarization. To investigate whether HMGN2 can affect NO production in NTM‐induced M1 polarization, HMGN2‐specific siRNA was transfected into macrophage RAW264.7 and MH‐S. As shown in Figure S2B, siRNA‐mediated HMGN2 knock‐down achieved 90% down‐regulation of HMGN2 expression in both cell lines compared to negative control. With using the NO production assay, we measured that the enhanced production of NO in HMGN2 silenced RAW264.7 was at 12 hours after infection, while HMGN2 silenced MH‐S displayed the increasing of NO production at 6 hours (Figure 3A). Furthermore, consistent to the NO production results, we observed that siRNA‐mediated knock‐down of HMGN2 unregulated the NO synthase iNOS mRNA and protein level in both cell lines from 3 to 12 hours (Figure 3B and C).

### 3.4 HMGN2 knock‐down enhanced M1‐related pro‐inflammatory cytokine expression

**Figure 4:**
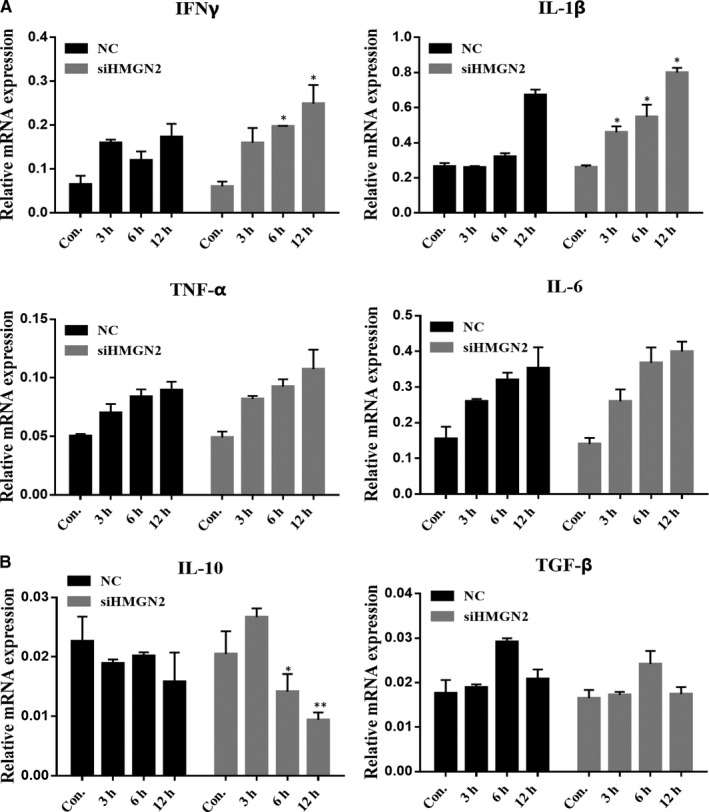
HMGN2 knock‐down up‐regulates pro‐inflammatory cytokines in NTM‐induced M1 macrophage polarization. RAW264.7 cells were transfected with siRNA‐HMGN2 and NC siRNA (scramble control) for 24 h and then incubated with *Ma.24* (MOI = 10:1) for 3, 6 and 12 h. A, The production of IFNγ; IL‐1β; TNF‐α; and IL‐6 was examined by RT‐qPCR *P* value was determined by *t* test. *Indicates significant difference (*P < .05; **P < .01) between NC‐infected and siHMGN2‐infected groups. B,The expression levels of IL‐10 and TGF‐β were determined by RT‐qPCR *P* value was determined by *t* test. *Indicates significant difference (*P < .05; **P < .01) between NC‐infected and siHMGN2‐infected groups. Results shown are representative of three independent experiments

In addition to the analysis of iNOS gene expression, we also investigated whether HMGN2 knock‐down can affect pro‐inflammatory cytokine (TNF‐α, IFNγ, IL‐1β and IL‐6) expression in macrophage after NTM infection condition. As shown in Figure 4A, HMGN2 knock‐down enhanced IFNγ expression in the infected RAW264.7 at 6 and 12 hours, and an enhanced expression of IL‐1β was exhibited at 3, 6 and 12 hours. Although we have not observed a significant increased expression for TNF‐α and IL‐6, an increased tendency was displayed after HMGN2 knock‐down in RAW264.7 with *Ma. 24* infection. At meanwhile, we analysed the expression of two anti‐inflammatory cytokines IL‐10 and TGF‐β which were well known as M2 markers. As shown in Figure 4B, *Ma.24* infection caused the down‐regulation of IL‐10 which was further decreased by HMGN2 knock‐down in macrophages, while we observed that there is no obvious influence by *Ma.24* infection and HMGN2 knock‐down on TGF‐β expression in macrophage.

### 3.5 HMGN2 knock‐down enhanced the activation of NF‐κB signalling pathway

**Figure 5:**
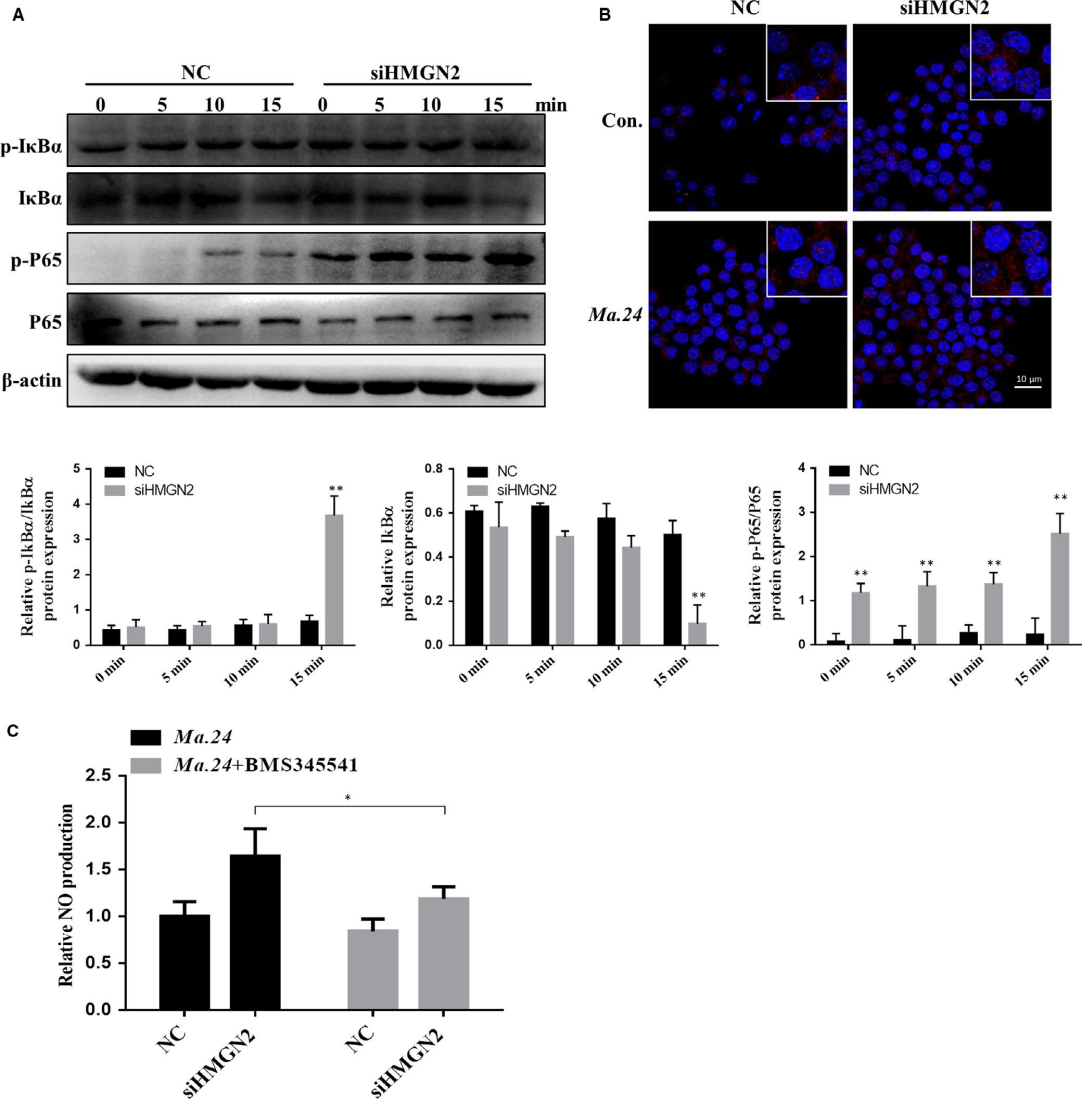
HMGN2 knock‐down enhances key M1 macrophage polarization signaling pathway NFκB. RAW264.7 cells were transfected with siRNA‐HMGN2 and NC siRNA (scramble control) for 24 h and then incubated with *Ma. 24* (MOI = 10:1) for 5, 10 and 15 mins. Western blot showing phosphorylated and total levels of IκBα, P65(A). B, The location of P65 was detected by immunofluorescence microscopy. P65 was labelled with TRITC and cell nucleus was stained by DAPI. C, siHMGN2 and NC RAW264.7 cells were treated with inhibitor BMS34541 for 24 h and then incubated with *Ma.24* (MOI = 10:1) for 12 h. The production of NO was examined by nitrate reduction method. *P* value was determined by *t* test. *Indicates significant difference (*P < .05) between BMS345541‐treated and untreated groups. Results shown are representative of three independent experiments

NF‐κB is regarded as a major immune‐regulating transcription factor to regulate the expression of iNOS gene and other pro‐inflammatory mediators during M1 macrophage polarization.^33^, ^34^, ^35^ At once activation of NF‐κB signalling, IκBα was phosphorylated and ubiquitination, further be subsequent proteasomal degradation, which leading to P65 phosphorylation and transfer to nuclear. We examined whether loss the function of HMGN2 could impact on *Ma.24*‐induced IκBα expression. As shown in Figure 5A, knock‐down of HMGN2 in macrophage strongly induced IκBα phosphorylation and then we observed a degradation of IκBα. Next, we observed that the phosphorylation of P65, a downstream signal cascade of NF‐κB pathway, was significantly enhanced in HMGN2 knock‐down cells (Figure 5A). Immunostaining study showed that HMGN2 knock‐down significantly promoted the p65 localization into the nucleus (Figure 5B). To further confirm HMGN2 regulates NO through NF‐κB signalling, we used IKK inhibitor BMS‐345541 in the silenced HMGN2 macrophage to inhibit the NF‐κB signalling and then measured the production of NO. As shown in Figure 5C, the HMGN2 knock‐down induced the production of NO was inhibited by BMS‐345541 in NTM‐infected macrophage.

Our previous studies showed that HMGN2 can inhibit ERK1/2 and JNK phosphorylation. As MAPK signalling plays an important role in modulation of the expression of pro‐inflammatory mediators expression in macrophages. Therefore, we also included analysis of JNK, P38 and ERK1/2 signalling pathways in the current study. As shown in Figure S2C, phosphorylation of JNK, P38 and ERK1/2 was activated by NTM infection from 10 to 15 minutes, which was further enhanced by HMGN2 knock‐down in macrophage.

### 3.6 HMGN2 was involved in IFNγ‐induced M1 macrophage polarization

**Figure 6:**
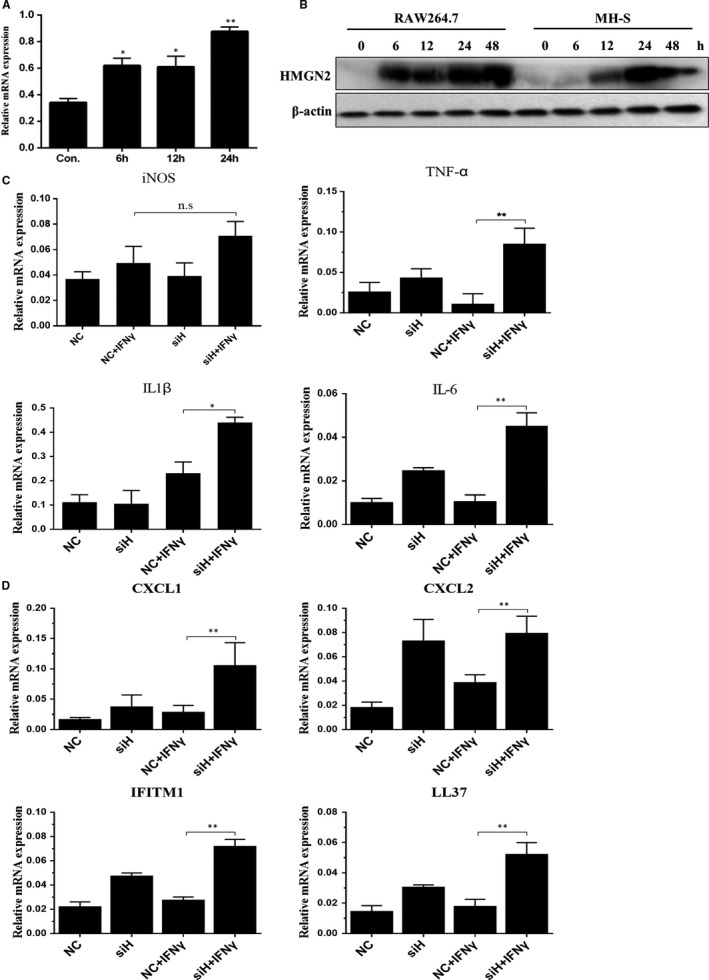
HMGN2 knock‐down enhances IFNγ‐induced M1 macrophage polarization. A, The transcription of HMGN2 in RAW264.7 treated with IFNγ at 6, 12 and 24 h was detected by RT‐qPCR. Results shown are representative of at least three independent experiments. *P* value was determined by *t* test. *Indicates significant difference (*P < .05) between treated and untreated groups. B, The expression of HMGN2 in RAW264.7 treated with IFNγ at 6, 12, 24 and 48 h was detected by Western blot. Results shown are representative of at least three independent experiments. C, Transcript levels of inflammatory factors genes were measured by RT‐qPCR for RNA isolated from RAW264.7 transfected with siRNA‐HMGN2 and NC siRNA responded to IFNγ‐treated cells for 24 h. *P* value was determined by *t* test. *Indicates significant difference (*P < .05, **P < .01) between NC‐treated and siHMGN2‐treated groups. Results shown are representative of at least three independent experiments. D, Transcript levels of IFITM1, LL37 and chemokines genes were measured by RT‐qPCR for RNA isolated from RAW264.7 transfected with siRNA‐HMGN2 and NC siRNA responded to IFNγ‐treated cells for 24 h. *P* value was determined by *t* test. *Indicates significant difference (**P < .01) between NC‐treated and siHMGN2‐treated groups. Results shown are representative of at least three independent experiments

As shown in above results, IFNγ, a well‐noted immune regulator to involve both in the induction of M1 macrophage polarization and anti‐NTM immune reaction in macrophage, was shown to be regulated by HMGN2. It is supposed that HMGN2 knock‐down enhanced M1 polarization were possibly and partially mediated by IFNγ. In order to explore this possibility, we investigated the role of HMGN2 in regulation of IFNγ‐induced M1 macrophage polarization. As shown in Figures 6A and B and S4A, the mRNA expression of HMGN2 was induced by IFNγ from 6 to 24 hours, and HMGN2 protein was induced by IFNγ from 6 to 48 hours. Then, we observed that IFNγ‐induced TNF‐α, IL‐1β and IL‐6 transcription were further promoted by knock‐down of HMGN2 compared with negative control group (Figure 6C). Although there is no significant change of iNOS production mediated by HMGN2 knock‐down, an obvious increase tendency was showed. In addition, we analysed antiviral gene expression IFITM1, antibacterial gene LL37 as well as chemokines CXCL1 and CXCL2 (Figure 6D), and our results exhibited a significant induction of IFITM1 and LL37. Chemokines CXCL1 and CXCL2 were induced by IFNγ treatment, which were also further up‐regulated HMGN2‐deficient macrophages. Collectively, these results indicated an essential role of HMGN2 in regulating IFNγ‐induced macrophage polarization.

### 3.7 Knock‐down macrophage HMGN2 reduces the survival of intracellular NTM

**Figure 7:**
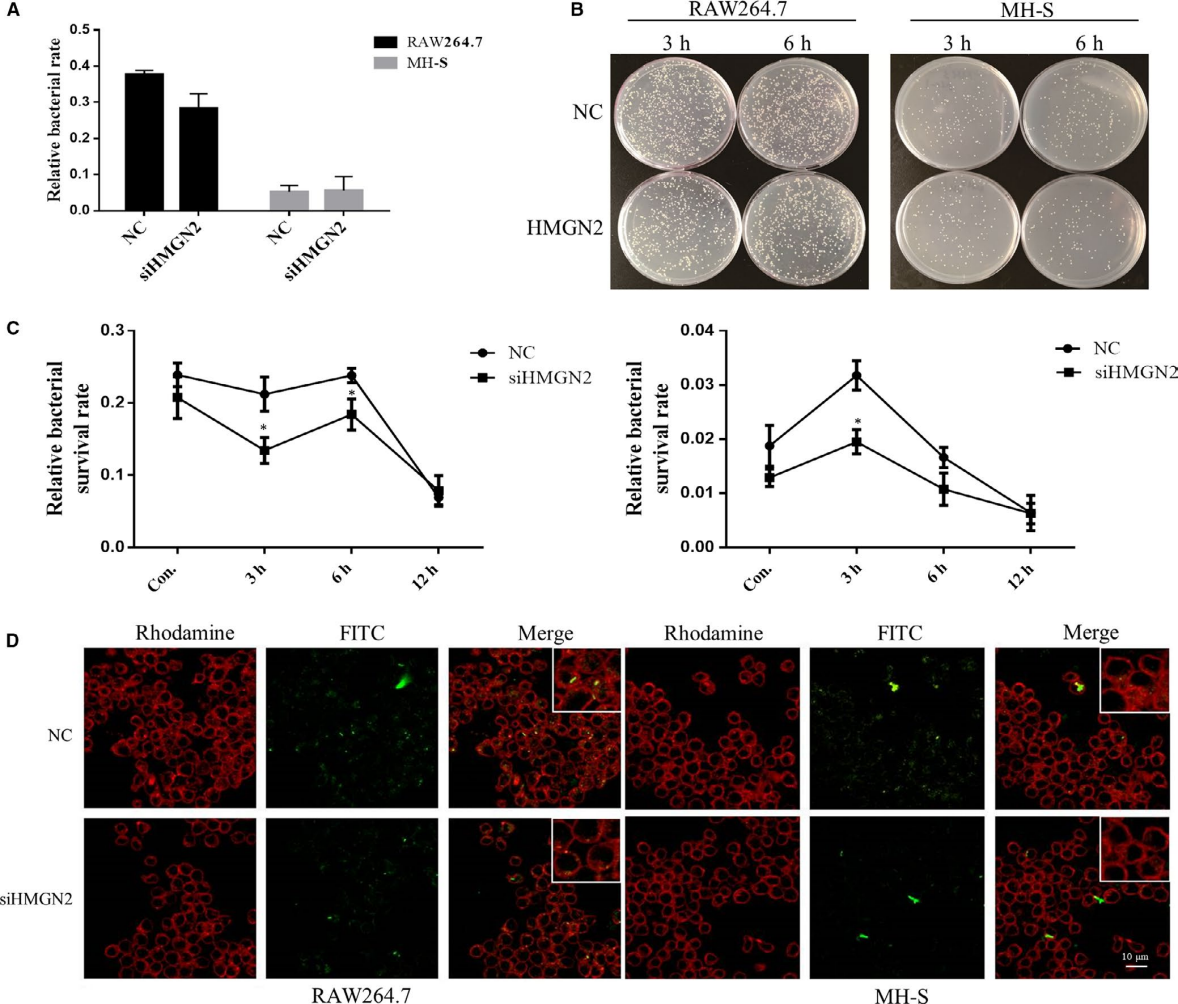
HMGN2 knock‐down affects NTM survival in macrophage. A, The survival of *Ma.24* in knock‐down of HMGN2‐infected macrophages 3 h was determined by colony‐forming unit assay and cultured macrophages after cleared the extracellular *Ma.24* for another 3, 6 and 12 h. Results shown are representative of at least three independent experiments. The colony‐forming unit showed the number of intracellular *Ma.24*, *and P* value was determined by *t* test (B and C). *Indicates significant difference (*P < .05) between NC‐infected and siHMGN2‐infected groups. D, *Ma.24*‐infected macrophages were showed by immunofluorescence microscopy. *Ma.24* was labelled with FITC

Finally, in order to investigate whether HMGN2‐regulated macrophage M1 polarization can manipulate the overall intracellular NTM, we preformed intracellular bacterial assay to examine the survival of *Ma.24* in macrophage with HMGN2 knock‐down. HMGN2‐deficient macrophages were co‐incubated with *Ma.24* at an MOI of 10:1 for 3 hours, and then the extracellular bacteria were killed by 100 µg/mL gentamicin and further the infected cells were cultured from 3 to 12 hours. As shown in Figure 7 B and C, knock‐down of HMGN2 significantly reduced *Ma.24* survival in RAW264.7 and MH‐S at the early stage of infection from 3 to 6 hours, while the invasion of *Ma. 24* had no significant change in both macrophages (Figure 7A). To further confirm these survival results, the FITC‐labelled *Ma.24* was applied to infect HMGN2 deficient macrophage. As shown in Figure 7D, we observed the higher fluorescent signal from the macrophage with HMGN2 knock‐down by using immunofluorescence microscope, which also indicated that HMGN2 knock‐down decreased the survival of NTM in macrophage. Collectively, our results proofed that HMGN2‐regulated M1 macrophage polarization can be functional relevance to its role in manipulation of NTM survival.

## 4 DISCUSSION

In the present study, we unravel a novel function of HMGN2 in regulation of classic activation of alveolar macrophages induced by NTM. We showed for the first time that HMGN2 was induced in NTM‐ and IFNγ‐mediated M1 polarization. RNAi‐mediated knock‐down of HMGN2 enhanced the expression of M1 marker genes such as iNOS, IFNγ, IL‐1β, CXCL1 and CXCL2 in M1‐polarized alveolar macrophage, suggesting a participation of HMGN2 in regulating anti‐NTM innate immune function of macrophage.

Macrophage as a major innate immune cell plays a critical role in bacterial infection pathogenesis.^8^ Mycobacteria are firstly taken up by exudate macrophages or tissue‐specialized macrophage, and then be cleared via innate immune defence responses to against mycobacterial infection. At the beginning of the present study, we found that NTM likewise other pathogens can stimulate a group of M1 macrophage polarization marker genes expression. Of note, activation of Toll‐like receptor (TLR) signalling through recognition of pathogen‐associated molecular patterns leads to the transcriptional activation of genes encoding for pro‐inflammatory cytokines, chemokines and anti‐bacterial and antiviral molecules. For instance, TLR2/6; TLR4 and TLR9 serve as recognition receptors for *Mycobacteria tuberculous* to activate the innate immunity response of host.^36^, ^37^ LPS together with IFNγ have been widely used for the induction of M1 polarization model to study regulatory molecular mechanism for polarization. In terms of the present study, we would suggest TLR2 might be one of the key receptors which is responsible for the recognition of NTM to initiate the M1 macrophage polarization. For example, Dong Min Shin et al^38^ showed that *Mycobacterium abscessus* stimulates the innate immune response of macrophage via the interaction between TLR2 and dectin‐1. Martine Gilleron et al^39^ have demonstrated that both *Mycobacterium smegmatis* and *Mycobacterium tuberculosis* H37Rv are able to activate TLR2 signalling pathway.

HMGN2 is the most conserved member of the HMGN family, involved in unfolding higher‐order chromatin structures and facilitating the transcriptional activation of various mammalian genes. Since 2005, we were the first to reported that the HMGN2 can functionally as an antimicrobial molecule to against bacteria,^28^ a number of our previous studies have demonstrated that HMGN2 not only contribute to the regulation of chromatin structures, but also play a role in the regulation of innate immune responses. For instance, we found that knock‐down of HMGN2 increases the internalization of *Klebsiella pneumoniae* into respiratory epithelial cells through the regulation of α5β1 integrin expression.^30^ HMGN2 involves in the miR‐155‐ and miR‐23a‐mediated manipulation of *Klebsiella pneumoniae* adhesion on human pulmonary epithelial cells.^40^ Our previous studies have mainly focused on the role of HMGN2 in epithelial cell function. HMGN2 has been shown as a quick responsive gene to bacterial infection in epithelial cell. Therefore, we speculated that the HMGN2 may also play a role in macrophage antibacterial infection. In the present study, we were particularly interested in the role of HMGN2 in NTM induced the polarization of macrophage. As we expected, the expression of HMGN2 was elevated in NTM‐polarized M1 macrophage at the early time‐point, which was not accompanied with a significant localization change of HMGN2. As discussed above, TLR2 signalling pathway might contribute to the induction of HMGN2 upon NTM infection in macrophage. In addition, NF‐κB binding sites were found in HMGN2 promoter region with the use of online analysis of GeneCard database, which are two essential transcription factors during M1 macrophage polarization. Therefore, it was possible that NF‐κB, which is known to be direct downstream transcription factor of TLR2, contributed to the induction of HMGN2.

In order to investigate the functional relevance of the NTM‐induced HMGN2 up‐regulation, we firstly analysed the production of NO and iNOS in HMGN2‐deficient macrophage. Interestingly, we found that HMGN2 knock‐down increased NO production and iNOS expression, which indicated that the induced HMGN2 was not required for the production of NO and iNOS expression, but rather negatively regulated the M1 polarization. Consistent to iNOS results, expression of pro‐inflammatory cytokines IFNγ and IL‐1β was also shown to be significantly enhanced by HMGN2 knock‐down, which further confirmed the negative role of HMGN2 in regulation of M1 macrophage polarization. iNOS, inducible nitric oxide synthase, is a family of enzymes catalysing the production of nitric oxide (NO) from L‐arginine. High levels of NO enable to react with superoxide leading to peroxynitrite formation, which defined the contribution of iNOS in host antimicrobial immunity.^41^ In the present study, it is likely that HMGN2 knock‐down enhanced the host defensin against to NTM by the induction of iNOS expression.

Next, we tried to explore by which M1 polarization signalling pathways were influenced by HMGN2 knock‐down in macrophage upon NTM infection. It has been reported that *Mycobacterium avium* and *Mycobacterium abscessus* can trigger NF‐κB signalling activation and then stimulate the innate immune responses in human peripheral blood mononuclear cells. Moreover, NF‐κB is one of the essential signalling pathways involved in M1 polarization; therefore, we asked whether HMGN2 knock‐down–mediated induction of M1 marker genes was depended on these two pathways. In the present study, we found that HMGN2 negatively regulated the activation of NFκB signalling characteristic with enhanced IkBα degradation, p65 phosphorylation and nuclear translocalization in *Ma.24*‐infected macrophages with HMGN2 knock‐down. Furthermore, we confirmed the HGMN2 knock‐down–mediated enhancement of NFκB activation contributes to the increased production of NO by using NFκB inhibitor. Additionally, *Mycobacterium massiliense*, belongs to the *Mycobacterium.abscessus*, has been reported to induce pro‐inflammatory cytokines (TNF‐α, IL‐6 and IL‐1β) dependent on JNK signalling not on ERK1/2 or p38 pathway in BMDMs.^42^ In our study, we found that HMGN2 knock‐down enhanced all the three signalling pathways, and we need further experiments to proof whether it was responsible for the HMGN2 regulated M1 markers genes expression.

IFNγ is the key cytokine involved in the protective role to against *Mycobacterium.tuberculosis* and NTM infection. The involved mechanism includes the induction of autophagy, a number of antimicrobial molecules, and pro‐inflammatory cytokines and chemokines. As discussed above, IFNγ was also well known for its role in inducing M1 macrophage polarization. As shown in Figure 4A, IFNγ expression was significantly enhanced by HMGN2 knock‐down in macrophage upon NTM infection, thereby it is likely that HMGN2 regulated M1 macrophage polarization via the IFNγ. To answer this question, we further investigated the regulatory role of HMGN2 in IFNγ‐induced M1 macrophage polarization. To our expectation, HMGN2 knock‐down also enhanced M1 markers gene expression which was consistent to function of HMGN2 in NTM‐induced M1 polarization. Additionally, we found that HMGN2 knock‐down increased expression of CXCL1, CXCL2, IFITM1 and LL‐37. Notably, CXCL1 and CXCL2 are the chemoattractant for neutrophils, which indicated a possible role of HMGN2 in neutrophil‐mediated inflammation. IFITM1 is a member of the IFITM family (interferon‐induced transmembrane protein) which is encoded by IFITM genes. It has been demonstrated that IFITM proteins can be the antiviral restriction factors for influenza It has been demonstrated that IFITM proteins can be as antiviral restriction factors for inhibiting influenza A virus replication, which suggested a possible contribution of HMGN2 in innate immunity. LL‐37 is a kind of cathelicidin antimicrobial peptide, which can be induced by IFNγ and serve a critical role in innate immune defence against *Mycobacterium tuberculosis* infection. These results also indicated a possible role of HMGN2 in regulating NTM infection. Lastly, to our expectation, HMGN2 regulated M1 macrophage polarization which contributes to the overall NTM survival in macrophages. HMGN2 knock‐down reduced the ability of NTM survival ability. Moreover, our previous studies showed that HMGN2 attenuated pyocyanin‐induced oxidative stress to inhibit *Pseudomonas aeruginosa* internalization in lung epithelium cells.^31^ Our data revealed that HMGN2 also involved in *uropathogenic Escherichia coli* (UPEC) infection in bladder epithelial cells via regulating autophagy. Therefore, HMGN2 is one of the important regulators of anti‐bacterial innate immunity in both epithelial and macrophages.

In conclusion, our study reported that both NTM‐ and IFNγ‐induced M1 alveolar macrophages expressed a high level of HMGN2. Further functional analysis revealed that HMGN2 can regulate both NTM‐ and IFNγ‐induced M1 polarization, and further impacted on the NTM survival in macrophages. The important M1 polarization signalling pathways NF‐κB were found to be relevant to HMGN2 regulated M1 polarization. Collectively, present study suggests an novel role of HMGN2 in anti‐NTM innate immunity, and HMGN2‐specific inhibition or interfering might thus represent a favourable approach for the treatment of NTM‐related infection.

## CONFLICT OF INTEREST

The authors declare no conflict of interests.

## AUTHOR CONTRIBUTIONS

XW, HR, JC, JL, YW and YH conceived the study, acquired the data, and interpreted and analysed the data. XW and SC wrote the manuscript and revised it critically for the important intellectual content. All authors approved the final version to be published.

## Data Availability

All data generated during the study are included in this article.
