# Supplementary figures and images for: HMGN2 regulates non‐tuberculous mycobacteria survival via modulation of M1 macrophage polarization
Source: J Cell Mol Med. 2019 Oct 9;23(12):7985–98. doi: 10.1111/jcmm.14599 (PMC6850944; doi:10.1111/jcmm.14599)

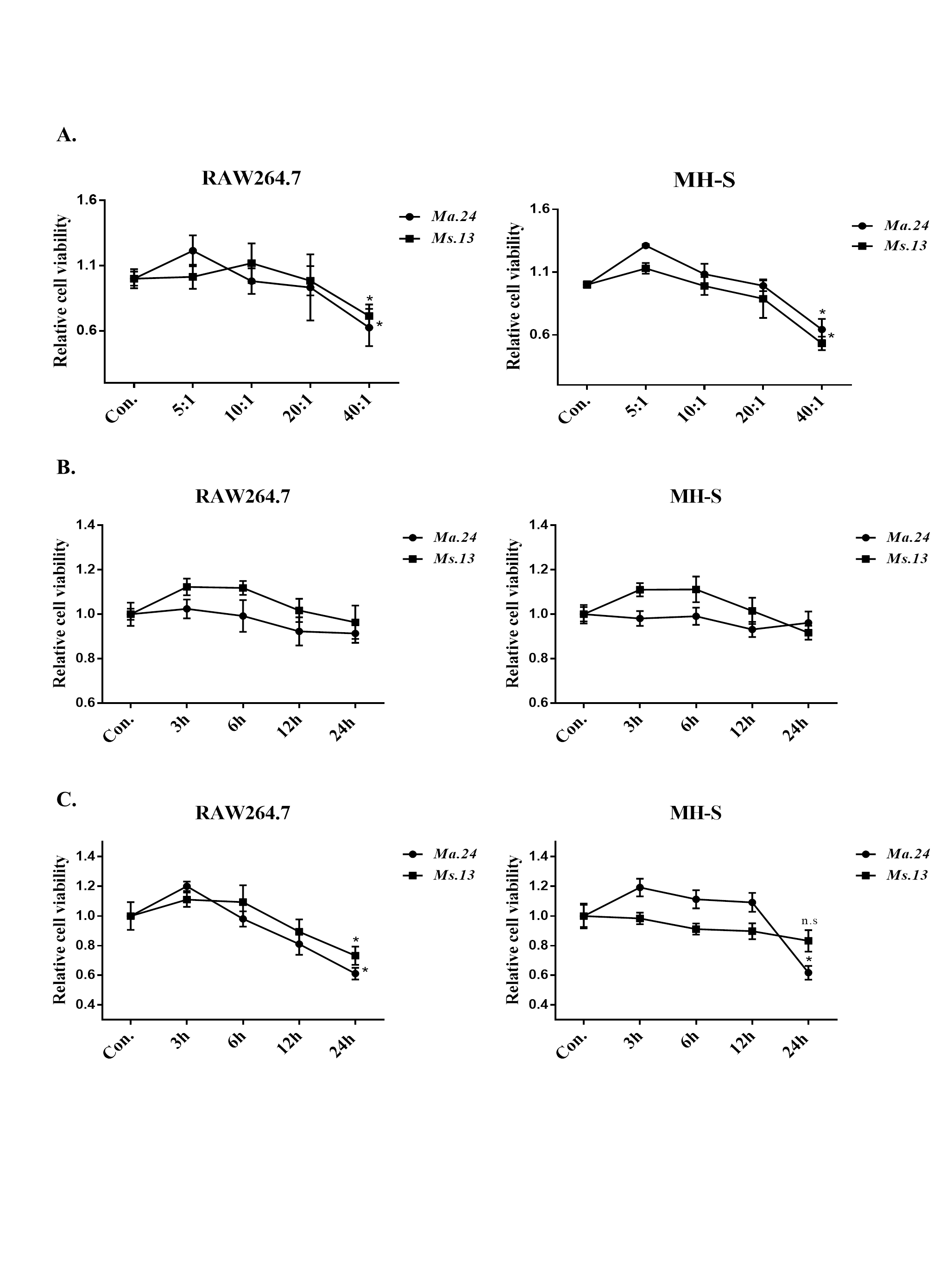

Supplement: Supplementary file 1 [file JCMM-23-7985-s001.TIF]

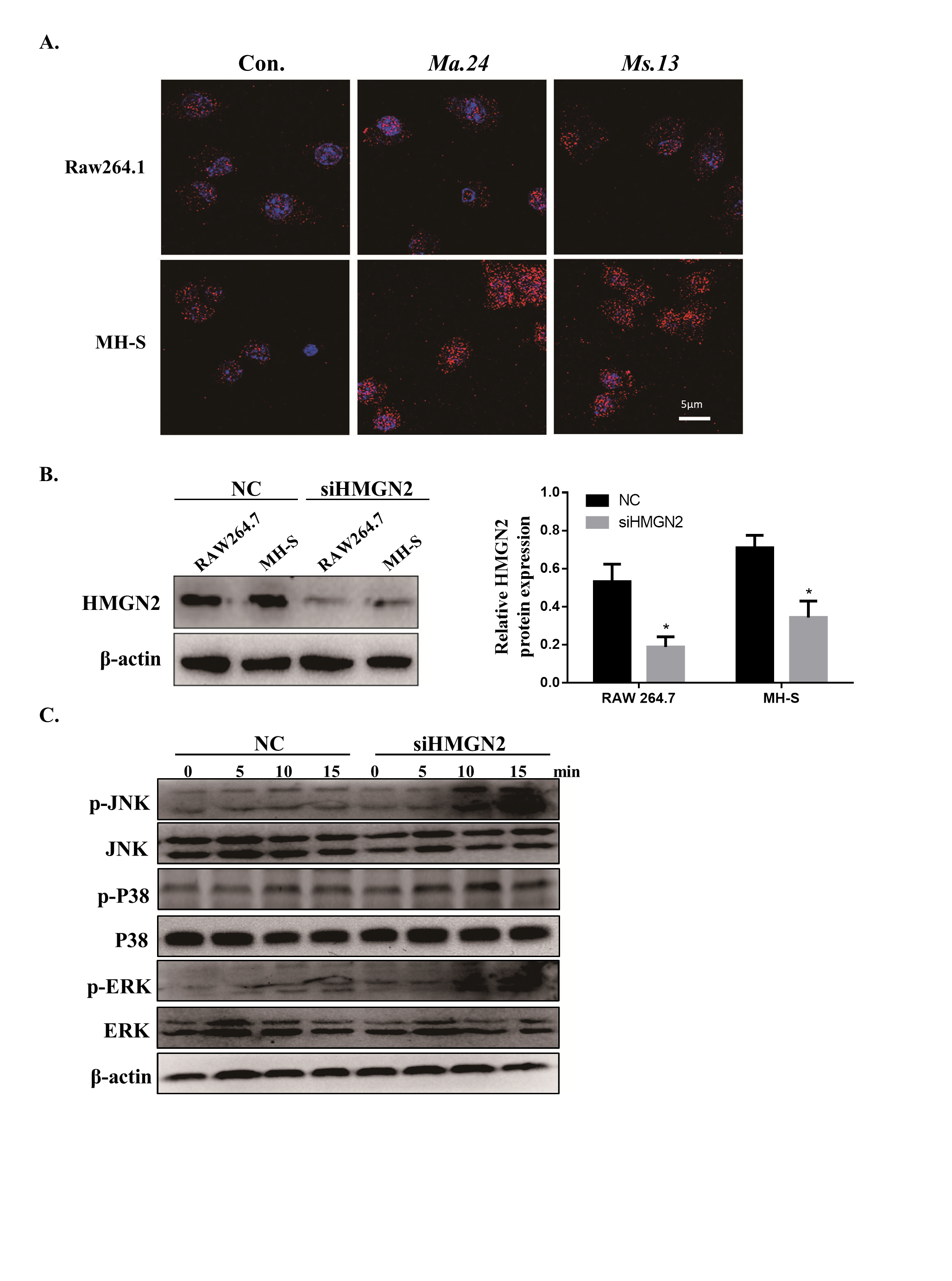

Supplement: Supplementary file 2 [file JCMM-23-7985-s002.TIF]

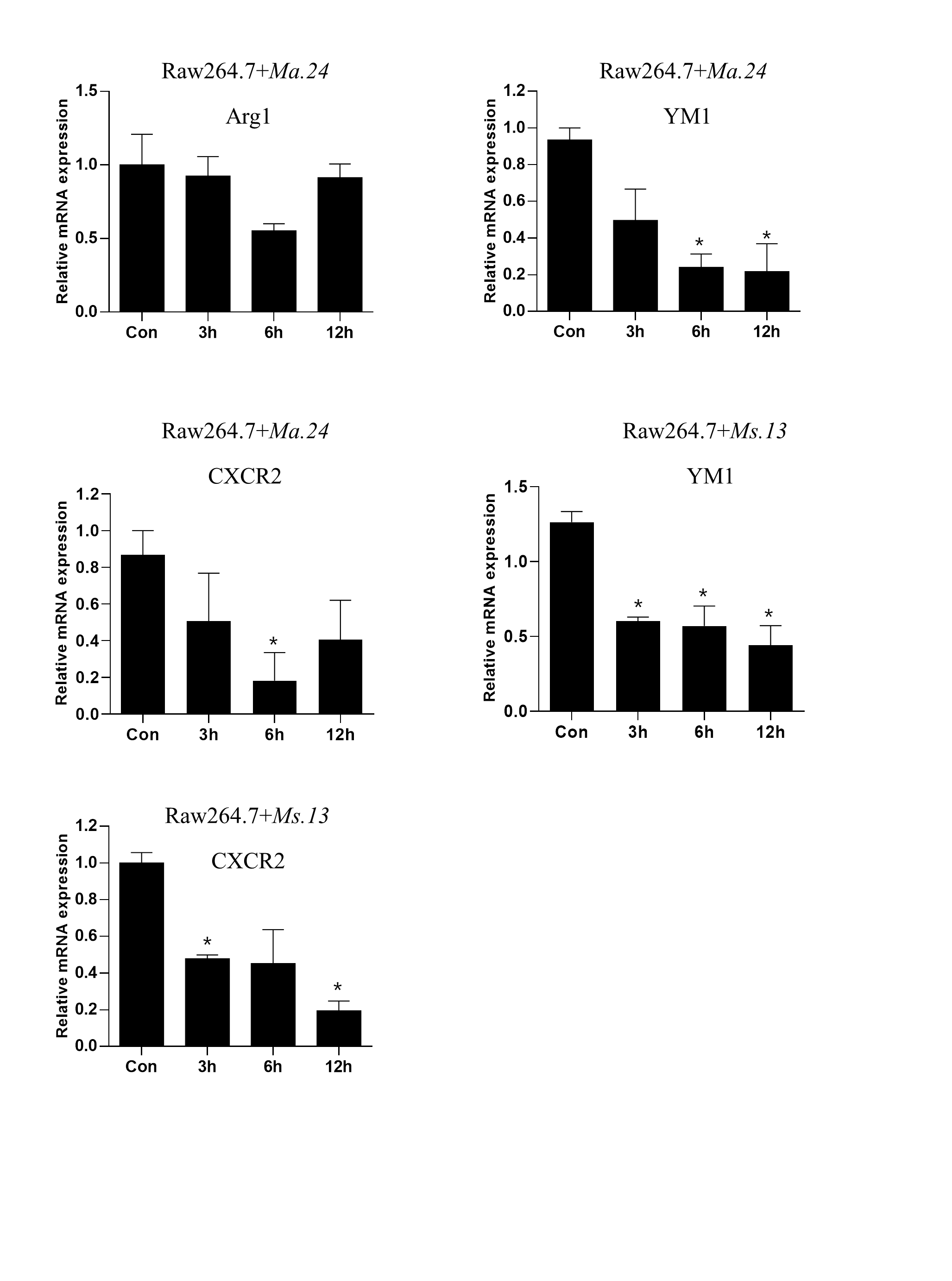

Supplement: Supplementary file 3 [file JCMM-23-7985-s003.TIF]

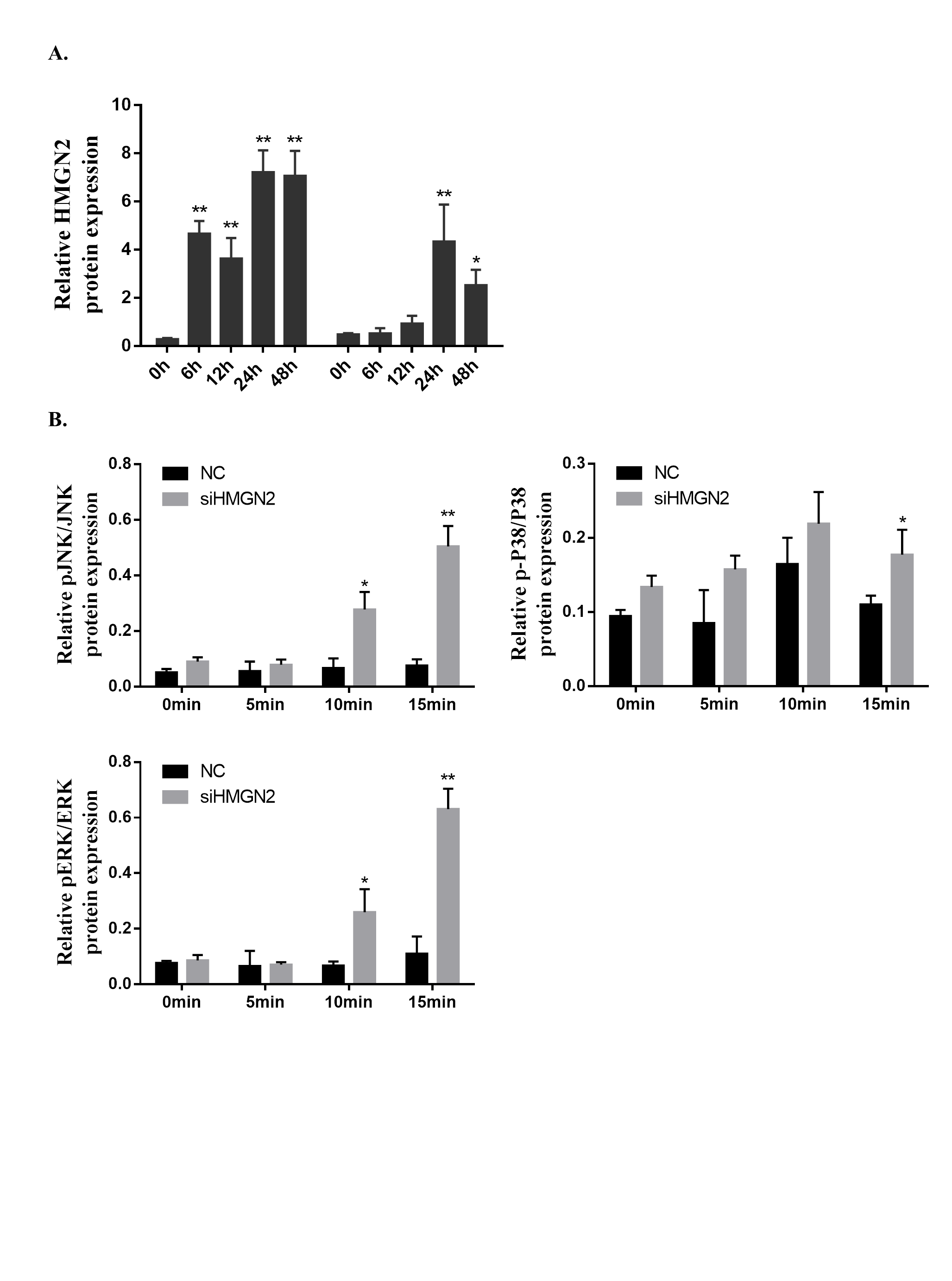

Supplement: Supplementary file 4 [file JCMM-23-7985-s004.TIF]
